# Trial steering committees for randomised controlled trials: updating and redeveloping guidance and terms of reference informed by current practice and experience

**DOI:** 10.1186/1745-6215-14-S1-P128

**Published:** 2013-11-29

**Authors:** EJ Conroy, S Lewis, A Lane, MR Sydes, J Norrie, G Murray, NL Harman, C Gamble

**Affiliations:** 1 Department of Biostatistics, University of Liverpool, Liverpool, UK; 2 Edinburgh MRC Hub for Trials Methodology Research, Edinburgh, UK; 3 Bristol Randomised Trials Collaboration Trials Unit, Bristol, UK; 4 MRC Clinical Trials Unit, London, UK; 5 MRC Clinical Trials Unit Hub for Trials Methodology Research, London, UK; 6 Centre for Healthcare Randomised Trials (CHaRT), Aberdeen, UK; 7 The Healing Foundation Cleft and Craniofacial Clincial Research Centre, University of Manchester, Manchester, UK; 8 Medicines for Children Research Network Clinical Trials Hub, Liverpool, UK

## 

The DAMOCLES project established a Data Monitoring Committee Charter which has been widely used for randomised controlled trials since 2005. As established within DAMOCLES, the DMC is typical advisory, making recommendations to another executive body; considered the Trial Steering Committee. No evidence-based Charter exists for TSCs to establish their role and functionality in RCTs.

The MRC Guidelines for Good Clinical Practice (1998) defines a three committee oversight structure: the day-to-day Trial Management Group, the DMC and the executive TSC. The document provides brief Terms of Reference for TSCs and represents the first attempt at guidance on TSC remit and structure. It is not known whether or how extensively this document has been used to inform TSC-like current roles and practice. There is acknowledged variation in practice in the UK and, moreover, internationally.

The aim of this project was to re-visit the MRC Terms of Reference and provide dedicated guidance on TSC remit and structure by producing a comprehensive Terms of Reference and Charter that will promote a systematic and transparent approach to the oversight of RCTs.

To inform this development, a survey to establish current practice and requirements within UK registered Clinical Trials Units has been undertaken. A cohort of published RCTs within medical journals and HTA monographs has been obtained to determine how TSC activities are currently reported and the literature reviewed to identify case studies of TSC activity in RCT conduct.

Results of this work will be presented with how these findings impact development of more complete guidelines.

